# Intracorneal Hematoma Showing Clinical and Dermoscopic Features of Acral Lentiginous Melanoma

**DOI:** 10.1155/2017/3509146

**Published:** 2017-05-08

**Authors:** Ugur Uslu, Franz Heppt, Michael Erdmann

**Affiliations:** Department of Dermatology, Friedrich-Alexander-University Erlangen-Nürnberg (FAU), Universitätsklinikum Erlangen, Erlangen, Germany

## Abstract

Intra- and subcorneal hematoma, a skin alteration seen palmar and plantar after trauma or physical exercise, can be challenging to distinguish from in situ or invasive acral lentiginous melanoma. Thus, careful examination including dermoscopic and histologic assessment may be necessary to make the correct diagnosis. We here present a case of a 67-year-old healthy female patient who presented with a pigmented plantar skin alteration. Differential diagnoses included benign skin lesions, for example, hematoma or melanocytic nevus, and also acral lentiginous melanoma or melanoma in situ. Since clinical and dermoscopic examinations did not rule out a malignant skin lesion, surgical excision was performed and confirmed an intracorneal hematoma. In summary, without adequate physical trigger, it may be clinically and dermoscopically challenging to make the correct diagnosis in pigmented palmar and plantar skin alterations. Thus, biopsy or surgical excision of the skin alteration may be necessary to rule out melanoma.

## 1. Introduction

Intra- and subcorneal hematoma, a skin alteration seen palmar and plantar after trauma or physical exercise (usually on the heel as* talon noir*), can be challenging to distinguish from in situ or invasive acral lentiginous melanoma [1–3]. Thus, careful examination including dermoscopic and histologic assessment may be necessary to make the correct diagnosis [1–3]. We here report a patient with a plantar intracorneal hematoma showing clinical and dermoscopic features of melanoma, leading to surgical excision of the skin alteration.

## 2. Case Presentation

**Figure 1:**
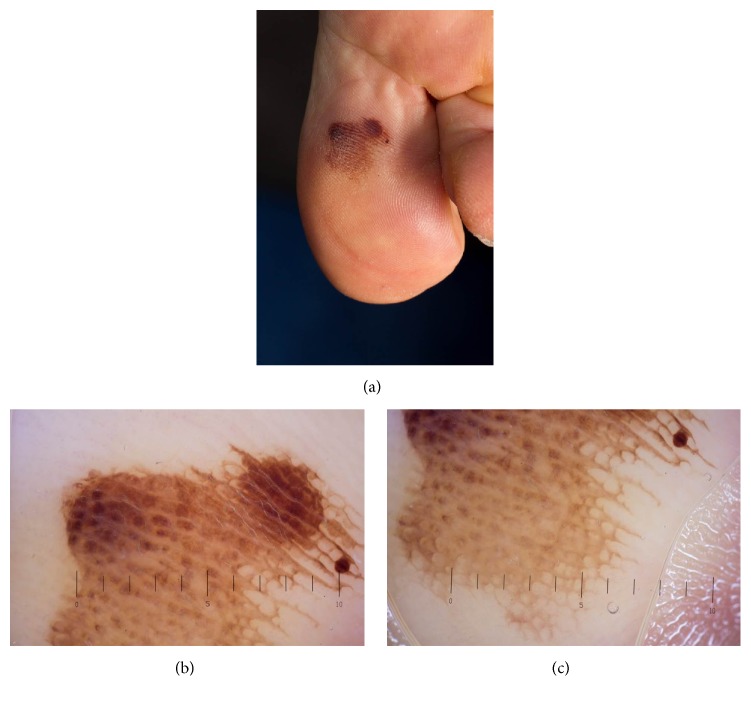
Clinical (a) and dermoscopic (b, c) features of the skin alteration. Notable is an asymmetric, irregular brown macula on the plantar side of the right hallux, approximately 15 mm in diameter (a). In dermoscopy, border irregularity and parallel-ridge pattern showing light and dark brown pigmentation with intersecting connection lines being seen (b, c).

**Figure 2:**
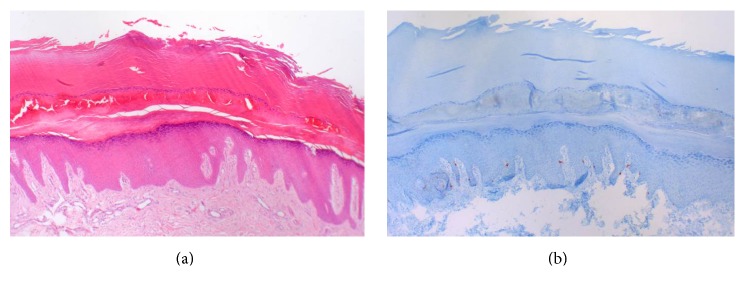
Histopathology (a) and immunohistochemistry (b) of the surgically removed skin alteration (original magnification ×40). Hematoxylin and eosin stain shows thickened orthokeratosis typical for acral skin, sampled stratum corneum with parakeratosis, serosanguinous fluid, and degenerated erythrocytes (a). Immunohistochemical stain for Melan-A revealed regular distribution of basal melanocytes without evidence of malignancy (b).

A 67-year-old healthy female patient without personal or family history of skin cancer presented for skin cancer examination, which revealed an asymmetric, irregular brown macula on the plantar side of the right great toe, approximately 15 mm in diameter (Figure 1(a)). Due to the localisation of the lesion, she had not noticed it before. Recent trauma, punctated blister, new shoes, and physical exercise were denied. Also, the patient did not take any anticoagulants. Dermoscopic examination revealed border irregularity and a parallel-ridge pattern showing light and dark brown pigmentation with intersecting connection lines and a partly veiled appearance (Figures 1(b) and 1(c)). Scraping test did not remove the pigmentation. Differential diagnoses included benign skin lesions, for example, hematoma or melanocytic nevus, and also acral lentiginous melanoma or melanoma in situ. Since clinical and dermoscopic examinations did not rule out a malignant skin lesion, surgical excision with primary closure under local anesthesia was performed. Histopathology revealed thickened orthokeratosis typical for acral skin, sampled stratum corneum with parakeratosis, serosanguinous fluid, and degenerated erythrocytes, consistent with an intracorneal hematoma (Figure 2(a)). Staining for Melan-A showed regular distribution of basal melanocytes without evidence of melanocytic neoplasia (Figure 2(b)). Annual skin cancer examination was recommended to the patient.

## 3. Discussion

Dermoscopic features of an intracorneal or subcorneal hematoma may mimic initial acral lentiginous melanoma or melanoma in situ [1, 4, 5]. Typical features of acral melanoma include, for example, colour variegation in a parallel-ridge pattern, border irregularity, and features of regression, which includes colour loss with a bluish-white veiled appearance [1, 4]. In hematoma lesions, reddish-black areas of homogenous pigmentation are typically observed, often accompanied by red-brown globules at the periphery, so-called satellite globules [1, 4]. In about 40% of hematomas, parallel-ridge pattern of pigmentation can also be found [1]. As our patient indeed showed clinical and dermoscopic features of both hematoma and melanoma and no adequate physical trigger was stated, surgical excision of the skin alteration was performed.

Ishihara et al. could show that 20 out of 22 acral melanocytic lesions which showed a parallel-ridge pattern in dermoscopy, also seen in 40% of hematomas [1], were indeed diagnosed with melanoma in situ [6]. A useful tool to distinguish between a hematoma and melanocytic neoplasia on the sole of the foot is a so-called scraping test [4]. In case of a hematoma, the pigmentation of the lesion can be partial or completely removed by gentle scraping off the stratum corneum [4]. Since pigmentation in a melanocytic lesion is never limited to the stratum corneum, the scraping test is negative. In some hematoma cases, however, removal of the pigmentation is not observed, like in our patient.

In summary, without adequate physical trigger, it may be clinically and dermoscopically challenging to make the correct diagnosis in pigmented palmar and plantar skin alterations. Thus, biopsy or surgical excision of the skin alteration may be necessary to rule out melanoma.
